# Case Report: S-100 negative primary esophageal malignant melanoma: surgical challenges and phenotypic evolution

**DOI:** 10.3389/fonc.2026.1786230

**Published:** 2026-04-13

**Authors:** Chenglin Fan, Hongbin Yin, Jingjun Zhu, Gaoyuan Sang, Ziyi Liang, Zhengzhe Xu

**Affiliations:** Department of Thoracic Surgery, The Affiliated Hospital of Yanbian University (Yanbian Hospital), Yanji, China

**Keywords:** 2-field lymphadenectomy, immunotherapy, phenotypic heterogeneity, primary malignant melanoma of the esophagus, S-100 negative

## Abstract

**Background:**

Primary malignant melanoma of the esophagus (PMME) is a rare malignancy associated with a dismal prognosis. Diagnosis typically relies on immunohistochemical markers such as HMB-45 and S-100. However, unlike its cutaneous counterpart, mucosal melanoma can present with an S-100 negative phenotype in a notable subset of cases, posing a significant diagnostic challenge. Furthermore, the optimal extent of lymphadenectomy (2-field vs. 3-field) remains controversial, particularly for elderly patients with limited physiological reserve.

**Case presentation:**

We report the case of a 64-year-old male presenting with progressive dysphagia. Endoscopy revealed a pigmented, polypoid mass in the middle esophagus. Due to severe abdominal adhesions from prior surgeries and the patient’s advanced age, the surgical team adopted a “safety-first” strategy, performing an Ivor-Lewis esophagectomy with 2-field lymphadenectomy. Postoperative pathology confirmed PMME (pT1bN0M0). Immunohistochemistry revealed a rare phenotype: HMB-45 positive (+) but S-100 negative (-). Despite adjuvant maintenance therapy with Toripalimab (a PD-1 inhibitor), the patient developed a rapid cervical lymph node recurrence four months postoperatively. Notably, the recurrent tumor cells exhibited partial S-100 positivity, indicating phenotypic heterogeneity and antigenic drift.

**Conclusion:**

This case highlights the potential diagnostic pitfall of S-100 negativity in PMME and the phenomenon of phenotypic switching during metastasis. For elderly patients where 3-field lymphadenectomy is deemed too risky, the high potential for cervical skip metastasis necessitates rigorous postoperative surveillance. The rapid disease progression despite immunotherapy underscores the urgent need to explore more effective multimodal treatment strategies.

## 1 Introduction

Primary malignant melanoma of the esophagus (PMME) is an exceptionally rare clinical entity, originating from melanocytes in the basal layer of the esophageal squamous epithelium (1) and accounting for only 0.1%–0.2% of all esophageal malignancies (2, 3). It is characterized by highly aggressive biological behavior and a poor prognosis (4). Currently, surgical resection remains the cornerstone of treatment. However, significant controversy persists regarding the optimal extent of lymphadenectomy—specifically, how to balance oncological radicality (McKeown/3-field) with physiological tolerance (Ivor-Lewis/2-field) (5).

The pathological diagnosis of PMME relies heavily on specific immunohistochemical markers, with S-100 and HMB-45 being the most sensitive indicators (6, 7). While the absence of S-100 expression is exceptionally rare in cutaneous lesions, a distinct proportion of mucosal melanomas lack this marker, frequently leading to misdiagnosis or missed diagnosis. Furthermore, compared to cutaneous melanoma, the efficacy of immune checkpoint inhibitors (ICIs) in mucosal melanoma is less established (8), and the interplay between lymph node dissection and the immune response requires further investigation.

Here, we report a rare case of PMME presenting with an initial S-100 negative phenotype. This case illustrates the surgical dilemma in an elderly patient with complex abdominal adhesions, the rapid development of cervical skip metastasis following 2-field lymphadenectomy, and the phenomenon of phenotypic antigen drift (S-100 re-expression) during disease progression.

## 2 Case description

### 2.1 Patient information

A 64-year-old male was admitted to our department in June 2022 complaining of progressive dysphagia for two weeks. He had a surgical history of hepatolithiasis and pyloric ligation, which resulted in extensive and severe abdominal adhesions. The patient denied any family history of tumors. A physical examination revealed no superficial lymphadenopathy, and a systemic skin examination excluded primary cutaneous melanoma.

### 2.2 Clinical findings and diagnostic assessment endoscopy and endoscopic ultrasound

**Figure 1:**
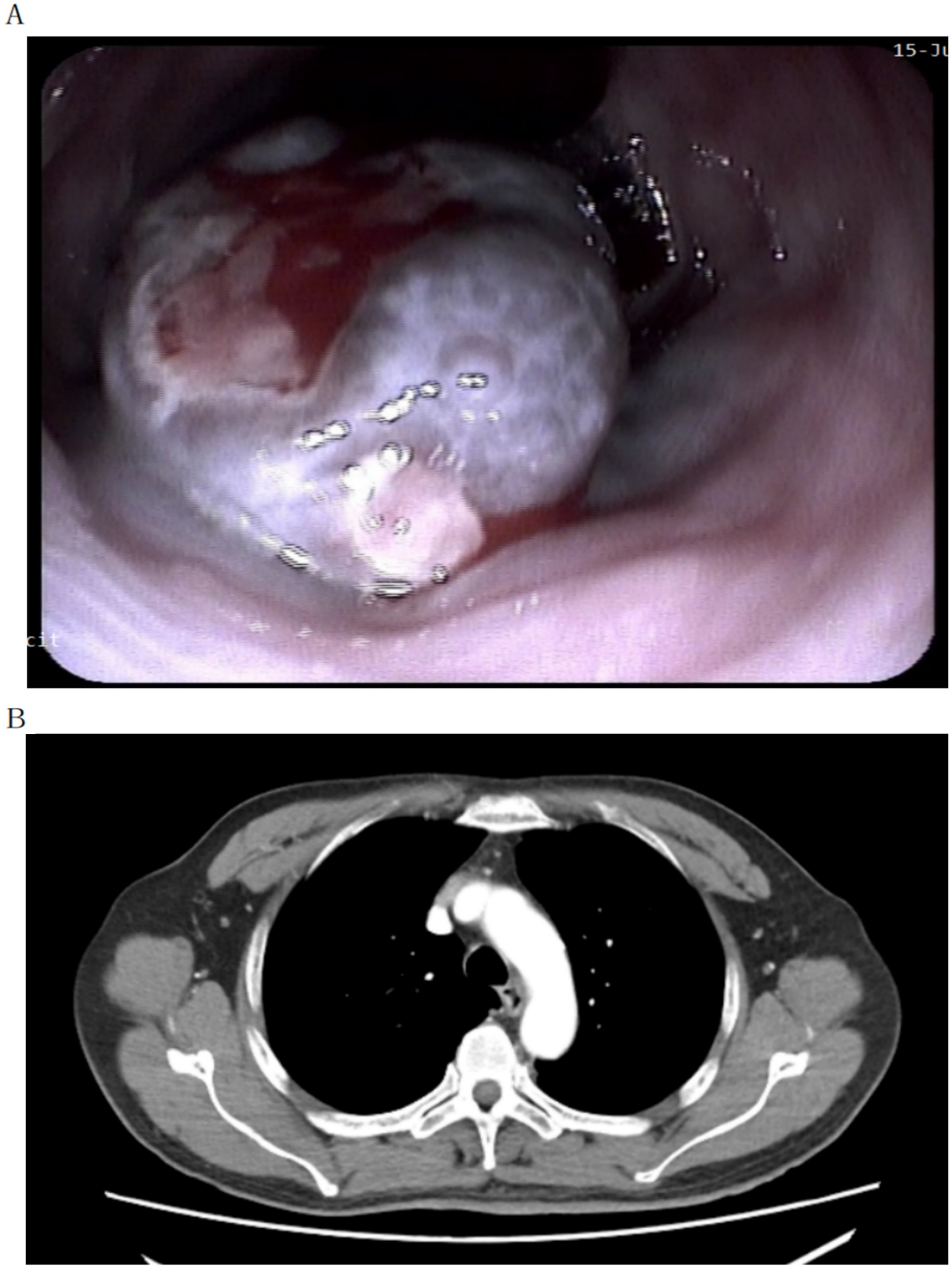
Diagnostic imaging findings. **(A)** Endoscopy and Endoscopic Ultrasound (EUS) revealing a longitudinal, blue-colored, polypoid mass (approx. 2.0 cm × 4.0 cm) located 27 cm from the incisors. **(B)** Contrast-enhanced CT of the chest showing thickening and enhancement of the middle esophageal wall without obvious mediastinal lymphadenopathy.

revealed a longitudinal, blue-colored, polypoid mass measuring approximately 2.0 cm × 4.0 cm, located 27 cm from the incisors (**Figure 1A**). The striking black-blue appearance of the mass initially raised clinical suspicion for primary malignant melanoma, esophageal melanosis, or a hemorrhagic poorly differentiated squamous cell carcinoma. Histopathological evaluation of the biopsy ultimately confirmed the melanoma. The lesion was accompanied by erosion and invaded the submucosa. Biopsy pathology suggested malignant melanoma. Contrast-enhanced CT of the chest showed thickening and enhancement of the middle esophageal wall, with no obvious lymphadenopathy in the mediastinum or abdomen (**Figure 1B**). Although PET-CT is the preferred modality for systemic staging of PMME to exclude occult metastases, it was not performed preoperatively due to the patient’s financial constraints. We acknowledge that this limited the preoperative assessment of micro-metastases to some extent (2, 3).

### 2.3 Therapeutic intervention

Following the PMME diagnosis, surgical resection was planned. The patient was 64 years old with moderate cardiopulmonary reserve. Furthermore, his history of multiple upper abdominal surgeries suggested a high risk of adhesions (intraoperative exploration later confirmed that adhesiolysis required 4 hours). Although neoadjuvant immuno-chemotherapy might improve response rates, recent studies suggest it may increase intraoperative cardiovascular instability and surgical complexity (9). To ensure perioperative safety and avoid the risks of prolonged anesthesia and potential recurrent laryngeal nerve injury, the surgical team decided against 3-field lymphadenectomy or neoadjuvant therapy. Instead, a “safety-first” strategy was adopted: Ivor-Lewis esophagectomy with 2-field lymphadenectomy (right thorax and upper abdomen).

### 2.4 Pathology and phenotypic characterization

**Figure 2:**
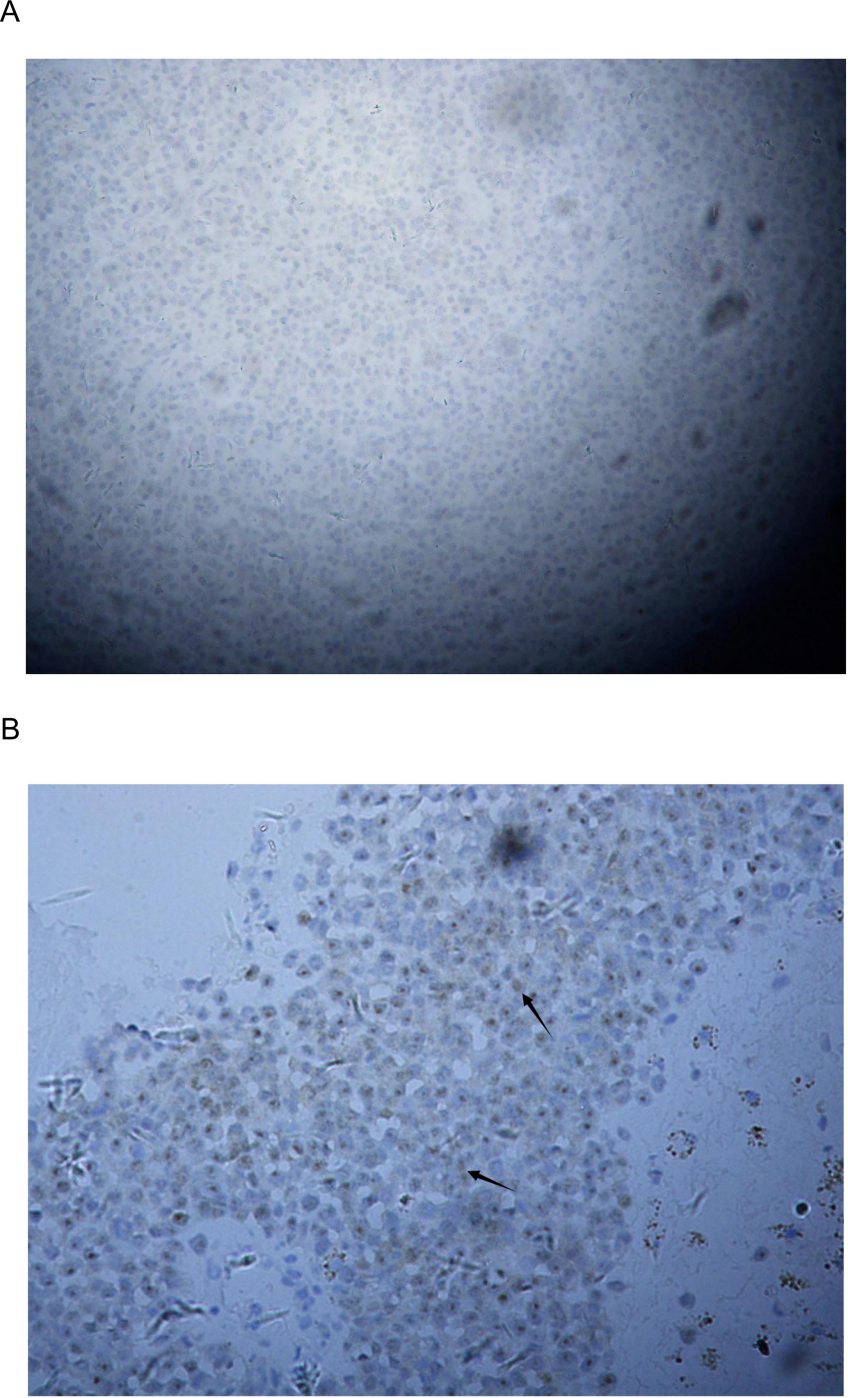
Histopathological and immunohistochemical characterization of the primary esophageal lesion. **(A)** negative for S-100 protein (Original magnification, ×200). **(B)** Focal positive staining for S100 was observed in both the nuclei and cytoplasm of the tumor cells (Arrows indicate areas of S-100 positivity). (Original magnification, ×400).

Postoperative pathology confirmed malignant melanoma of the esophagus, infiltrating the submucosa with vascular cancer emboli. Surgical margins were negative. A total of 11 lymph nodes were dissected from the subcarinal (Station 7), perigastric (Stations 17-19), and left gastric artery (Station 20) regions; all were negative for metastasis (0/11). Immunohistochemistry (IHC) of the primary tumor showed a distinct phenotype: HMB-45 (+), Vimentin (+), Ki-67 (80%+), but S-100 (-) (**Figure 2A**). The pathological stage was pT1bN0M0 (AJCC 8th Edition).

### 2.5 Follow-up and outcomes

**Figure 3:**
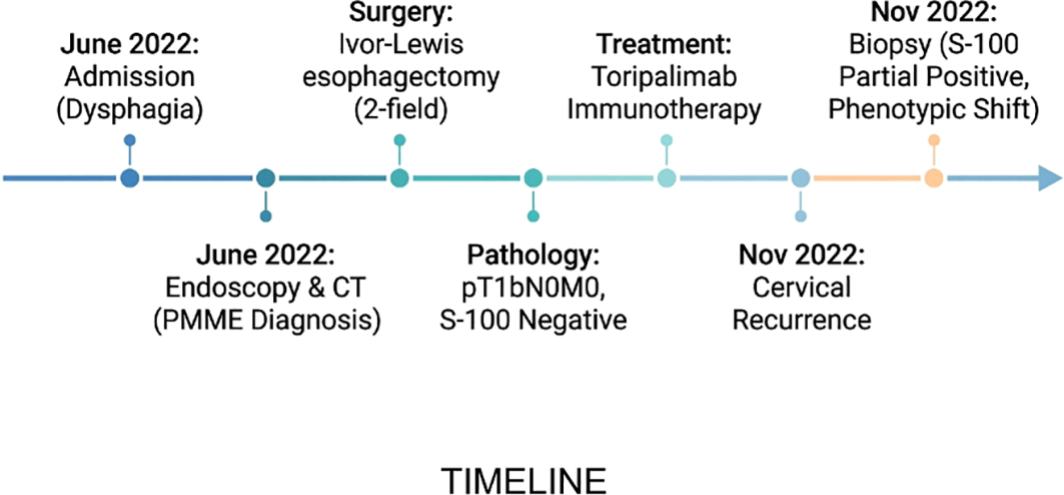
Timeline of the patient’s diagnosis, treatment, and disease progression.

Given the patient’s postoperative frailty and poor tolerance to the side effects of chemoradiotherapy, standard adjuvant chemotherapy was not administered. Therefore, the patient received maintenance immunotherapy with Toripalimab (a PD-1 inhibitor) (8). Despite this, the patient presented with a right cervical mass 4 months postoperatively (November 2022). Needle biopsy confirmed metastatic melanoma. Strikingly, IHC of the recurrent lesion indicated that S-100 expression had partially recovered (partial positive), suggesting phenotypic evolution of the tumor (10) (**Figure 2B**). A timeline of the case is presented in **Figure 3**.

## 3 Discussion

### 3.1 Diagnostic pitfalls: S-100 negativity and phenotypic heterogeneity

S-100 is generally a highly sensitive marker for melanoma. While its absence is exceptionally rare in cutaneous lesions, S-100 negativity actually occurs in a recognized subset of mucosal melanomas. Even so, the S-100-negative primary tumor in our patient still presented a major diagnostic trap, likely indicating a dedifferentiated state (11). Interestingly, the cervical recurrence later showed focal, heterogeneous S-100 re-expression. This phenotypic shift highlights how unstable these melanoma cells can be. It is still unclear if this re-expression was a spontaneous adaptation to the metastatic microenvironment (12) or a direct result of selective pressure from toripalimab therapy. Practically speaking, this means clinicians cannot rely on a single protein to diagnose PMME; a broader immunohistochemical panel, including HMB-45 and Melan-A, is absolutely necessary (13).

### 3.2 Surgical dilemmas: resection extent and risk trade-offs under uncertainty

Even though the primary tumor was pT1bN0M0, endoscopic en bloc resection was not a viable option. The patient had progressive dysphagia from a 2.0 × 4.0 cm polypoid mass; attempting endoscopic mucosal or submucosal dissection in this context carries a massive risk of severe postoperative strictures. Moreover, PMME is highly aggressive—often spreading laterally under the mucosa and invading blood vessels early on—so definitive anatomical resection was the only safe way to ensure negative margins.

When it came to the surgery itself, the ideal extent of lymph node dissection remains controversial. Our patient was clinically and pathologically N0 in the chest and abdomen, yet rapidly developed a cervical recurrence after a two-field dissection. This clearly illustrates mid-esophageal PMME’s tendency for skip metastasis (14). A key limitation here is that financial constraints prevented a preoperative PET-CT. Without that baseline, we cannot be sure if the cervical node was a true *de novo* skip metastasis or simply the progression of an undetected micrometastasis. Because of this ambiguity, we cannot definitively conclude that a two-field dissection is always inadequate for PMME. For this specific elderly patient with severe abdominal adhesions, a three-field clearance was simply too dangerous. The two-field approach was a necessary compromise to get him through the surgery safely (15). The main lesson is that if you have to perform a limited dissection on a high-risk candidate, you must follow up with aggressive surveillance of the undissected lymph node regions.

### 3.3 Limitations of immunotherapy, molecular profiling, and future directions

We know PMME resists conventional chemoradiotherapy, but our patient also progressed incredibly fast while on toripalimab (16). This primarily points to the fact that mucosal melanomas simply have higher intrinsic resistance to single-agent PD-1 inhibitors than cutaneous subtypes (8). As a secondary, more speculative point, the extensive lymph node dissection might have actually hindered the immunotherapy by clearing out the draining nodes needed for T-cell priming (17).

Looking back, a major gap in our management was skipping the BRAF V600 or c-KIT mutation tests. At the time of admission, all clinical focus was entirely on relieving his esophageal obstruction and dealing with the severe abdominal adhesions, so systemic molecular profiling was put on hold until the recurrence. In hindsight, for a frail patient who could not tolerate standard adjuvant chemoradiotherapy, finding an actionable BRAF V600 mutation would have opened the door to targeted therapy—a much better-tolerated systemic option.

Ultimately, the rapid progression and phenotypic shift in this case show that single-agent checkpoint inhibitors are just not enough. Moving forward, managing these highly aggressive tumors will require routine baseline molecular profiling alongside surgical planning, plus a serious evaluation of neoadjuvant therapies or multi-target combination regimens.

## 4 Conclusion

This case underscores the complexity of managing PMME. First, S-100 negativity does not exclude melanoma, and phenotypic drift can occur during metastasis. Second, while 2-field lymphadenectomy is a safer option for high-risk elderly patients, it leaves the cervical region vulnerable to skip metastasis, necessitating strict monitoring. Finally, single-agent immunotherapy may be insufficient for such aggressive phenotypes, highlighting the urgent need for novel multimodal treatment approaches.

## Data Availability

The original contributions presented in the study are included in the article/supplementary material. Further inquiries can be directed to the corresponding author.
